# Grey Matter Hypertrophy and Atrophy in Early-Blind Adolescents: A Surface-Based Morphometric Study

**DOI:** 10.1155/2022/8550714

**Published:** 2022-05-03

**Authors:** Fen Hou, Hengguo Li, Ping Li, Hongrong Shen, Yu Yang, Bo Li, Long Qian, Yang Fan, Hai Li, Gangqiang Hou, Wentao Jiang, Zhifeng Zhou, Xia Liu

**Affiliations:** ^1^Department of Radiology, First Affiliated Hospital of Hunan University of Chinese Medicine, 95 Shaoshan Middle Road, Yuhua District, Changsha 410007, China; ^2^Medical Imaging Center, The First Affiliated Hospital of Jinan University, Guangzhou 510632, China; ^3^3GE Healthcare, Beijing, China; ^4^Beijing Intelligent Brain Cloud, Inc, Beijing, China; ^5^Neuropsychiatric Imaging Center, Shenzhen Kangning Hospital, Shenzhen Mental Health Center, Shenzhen 518000, China

## Abstract

**Objective:**

This study is aimed at exploring the regional changes in brain cortical morphology (thickness, volume, and surface area) in the early-blind adolescents (EBAs) by using the surface-based morphometric (SBM) method.

**Methods:**

High-resolution structural T1-weighted images (T1WI) of 23 early-blind adolescents (EBAs) and 21 age- and gender-matched normal-sighted controls (NSCs) were acquired. Structural indices, including cortical thickness (CT), cortical volume (CV), and surface area (SA), were analyzed by using FreeSurfer software, and the correlations between structural indices and the blindness duration were computed by Pearson correlation analysis.

**Results:**

Compared to controls, EBAs had significantly reduced CV and SA mainly in the primary visual cortex (V1) and decreased CV in the left vision-related cortices (r-MFC). There were no regions that EBAs had a significantly larger CV or SA than NSCs. EBAs had significantly increased CT in the V1 and strongly involved the visual cortex (right lateral occipital gyrus, LOG.R) and the left superior temporal gyrus (STG.L), while it had decreased CT in the left superior parietal lobule (SPL.L) and the right lingual gyrus (LING.R). Additionally, no correlation was found between cortical morphometric measures and clinical variables in the EBA group.

**Conclusions:**

SBM is a useful method for detecting human brain structural abnormalities in blindness. The results showed that these structural abnormalities in the visual cortex and visual-related areas outside the occipital cortex in the EBAs not only may be influenced by neurodevelopment, degeneration, plasticity, and so on but also involved the interaction of these factors after the early visual deprivation.

## 1. Introduction

Blindness provides a rare model to explore the impacts of visual experience on the structural and functional organization of the human brain [1–5]. A number of studies have investigated brain structural abnormalities in early-blind people. These findings include changes of the local brain structures, such as significantly volumetric atrophy of gray matter [6–11], decreased SA [12, 13], increased CT [12–19], and impaired white matter integrity [20], as well as alterations of the brain structural networks, such as decreased network efficiency in the blind individuals [21].

However, most of them were based on volumetric measures, using voxel-based morphology (VBM) or deformation-based morphometry (DBM), which may conceal fine anatomic details in other features. Several VBM [6–8] and DBM [10] studies have observed significant volumetric atrophy of gray matter (GM) throughout the visual cortex in early-blind (EB) subjects [6–8, 10]. A more effective morphometry analysis tool than VBM and DBM is the SBM method which provides fine anatomic details in other features, such as CT and SA, and profoundly probe determining factors of CV approximated by multiplying CT by SA [22]. Previous SBM studies of blindness have shown decreased CV and SA [12, 13] and increased CT [12–19] in the occipital lobe. These changes may be accountable for following factors: neurodevelopment, degeneration, and plasticity [23, 24]. Previous research [25] indicated that degeneration and neurodevelopment may cause axonal damage and synapses interruption, which could lead to the reduced CV, SA, and CT of the visual cortex in the blind. However, other researches [26–28] concluded that plasticity and neurodevelopment may cause strengthened subcortical and corticocortical connections and reduced synaptic pruning, which could lead to the increased CV, SA, and CT in the visual cortex. From these previous results, we learned that these major factors, namely, neurodevelopment, degeneration, and plasticity, may influence differently on determining measures such as CV, SA, and CT. Therefore, these measures may change in even opposite directions (like a decreased CV or SA and an increased CT) under the interactions of these factors. However, the interactions of these factors remain to be not clearly understood.

In addition, these early blindness studies focused on the structural reorganization of the visual cortex, but the visual-related areas outside the occipital cortex in the blind have few been reported. Previous MRI studies of blindness reported reduced cortical thickness in the visual-related areas outside the occipital cortex (anterior STG and SPL) and increased cortical thickness in the visual cortex [12, 17]. Thus, whether the alteration mechanism in the thickness of the nonvisual cortex is as same as the visual cortex remains to be determined. Furthermore, it is interesting to explore the impact of the critical developmental period on the structural reorganization of these regions and the correlation between these structural indices and the blindness duration.

To elucidate these questions, in this study, we investigated cortical morphology (CT, CV, and SA) alterations in the visual cortex and visual-related areas outside the occipital cortex in the EBAs using a surface-based morphometric method [29]. We aimed to investigate the major factor(s) (neurodevelopment and/or degeneration and/or plasticity) how to play an interactive role in structural reorganization and possible neurological mechanism of structural reorganization in the blind.

## 2. Materials and Methods

### 2.1. Participants

Twenty-three EBAs (loss of sight at birth or within 1 year, 8 female, range = 11-18 years, mean age ± S.D.: 14.80 ± 2.07 years) were recruited from the Guangdong Province Blind School. Twenty-one NSCs (normal-sighted controls, NSCs) of volunteers (10 female, range = 11-19 years, mean age ± S.D.: 14.56 ± 2.59 years) participated in the study. Analysis of two-sample *T*-test did not indicate any significant difference in age (*t* = 0.33, *P* = 0.74), and a chi-squared test did not reveal a gender effect (*X*^2^ = 0.75, *P* = 0.39) between the EBA and NSC groups. Causes of blindness included retinopathy of prematurity, congenital retinal lesions, congenital glaucoma, and congenital cataracts. All participants met the following inclusion criteria: (1) right-handedness, (2) no history of neurological or psychiatric diseases and identifiable MRI normal structural brain, and (3) normal hearing. The study was approved by the Ethics Committee of the First Affiliated Hospital of Jinan University, and all subjects and their guardians signed a written informed consent form before undergoing the MRI examinations.

### 2.2. Data Acquisition

High-resolution 3D T1-weighted BRAVO images were obtained on a 3.0-Tesla MR scanner (Discovery MR750 System; General Electric, Milwaukee, WI, USA), fitted with an 8-channel head coil. The parameters were as follows: flip angle = 12°, TR = 8.2 ms, TE = 3.2 ms, FOV = 256 × 256 mm^2^, 256 × 256 matrix, slice gap = 0 mm, slice thickness = 1 mm, and 172 slices in the axial plane. Total scan time was 3 min 17 sec. During this MR scan, the subject's head was fixed using several foam cushions to minimize head motion.

### 2.3. Image Analysis

3D T1-weighted MRI data were processed and analyzed using FreeSurfer V6.0 [30, 31] (http://surfer.nmr.mgh.harvard.edu/) with a standard cross-sectional pipeline. Starting from nonuniform intensity normalization, removal of nonbrain tissue, transformation to Talairach space, skull stripping, segmentation into white-matter (WM) and gray-matter (GM), and tessellation into the WM and GM boundary, an initial surface was constructed. This surface was used to reconstruct the final cortical surface after smoothed, inflated, and automated topology correction. The cortical thickness measurements were produced by calculating the distance between these surfaces at each point across the cortical mantle [32]. The cortical thickness was compared node by node, and the statistical results were visualized by creating an average template and registering the cortical surface for each subject to it by a surface-based registration method [30, 31]. The cortex was parcellated, and the means of CT, CV, and SA were obtained at each point on the reconstructed surface. Finally, a heat kernel (10 mm width) was used to smooth the data of CT, CV, and SA to improve the normality of the data.

### 2.4. Statistical Analysis

Firstly, the obtained structural indicators were compared between groups based on vertices. For each hemisphere, the General Linear Model (GLM) with vertex-wise analyses of surface morphometric measurements, including CT, CV, and SA, was performed in EBA and NSC groups. Statistical maps were generated using FreeSurfer's Query, Design, Estimate, Contrast (QDEC) interface. Age and gender were introduced in the model as nuisance factors. Finally, multiple comparisons were corrected with Monte Carlo Simulation using a vertex-level *P* value set at <0.01, and cluster-level threshold *P* < 0.05. In addition, the Statistical Package for the Social Sciences (SPSS) software (version 23.0; IBM Corporation, NY, USA) was applied in all demographics analysis and the correlation analysis between cortical morphometric measures and subjects' age in both groups. Simultaneously, the correlation between cortical morphometric measures and blindness duration in the EBA group was examined. A two-tailed value less than 0.05 was considered statistically significant.

## 3. Results

**Figure 1:**
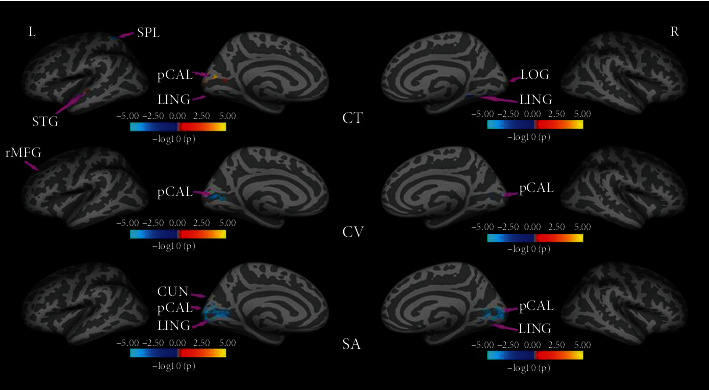
The significant altered cortical morphometry regions in EBAs when compared with the NSC group on CT, CV, and SA.

Compared to controls, EBAs had significantly increased CT in the left pericalcarine (pCAL.L), left cuneus (CUN.L), left superior temporal gyrus (STG.L), and right lateral occipital gyrus (LOG.R), while they had decreased CT in left superior parietal lobule (SPL.L) and right lingual gyrus (LING.R). In terms of the CV, EBAs had significant CV loss in bilateral pCAL and left rostral middle frontal gyrus when comparing with NSCs, and there were no CV increased regions found in EBAs. Regarding the SA, EBAs had significantly reduced SA in bilateral pCAL and LING and left cuneus (CUN.L) compared to NSCs. No larger SA was observed in EBAs in comparison to NSCs. The detailed data are summarized in Table 1 and illustrated in Figure 1. Additionally, no significant differences in the demographic characteristics were noted and no correlation was found between cortical morphometric measures and clinical variables in both groups (*P* > 0.05).

## 4. Discussion

### 4.1. Morphological Alteration in the Primary Visual Cortex (V1) and Vision-Related Cortices (Left Rostral Middle Frontal Gyrus, L-r MFC)

Our findings revealed significantly decreased CV and SA and increased CT in the V1 in the EBA group, which are similar to previous studies [12, 13]. These findings demonstrated that the development of V1 is strongly dependent on early visual experience [21, 25, 33, 34]. The following factors may affect the morphological alteration of the V1 during the developmental period. First, axonal degeneration is a key factor causing a reduced SA of the V1 in the early blind [25]. However, our finding of SA loss in the V1 of EBAs cannot completely be explained by axonal degeneration, which may be associated, at least to some degree, with visual loss leading to neurodevelopmental retardation during the developmental stage since no significant negative correlation was found between SA and blindness duration within the EBA group [12, 13]. Second, the increase of CT in the V1of EBAs cannot be explained by disuse atrophy and crossmodal plasticity at the adolescence stage, since there was no significant correlation found between CT and blindness duration within the EBA group. However, the increase of CT in the V1 may be attributed to the loss of normal neurodevelopment, which induces the reduction of synaptic pruning [35]. In addition, a thicker V1 is not found in the subjects who became blind in adulthood, whose V1 CT showed no difference from the sighted controls [33]. According to the microscopic neuroanatomical studies, the synaptic density in the human V1 is largest in the first postnatal year and gradually drops to the adult level during adolescence [36]. The disruption of the normal pruning process in EB, however, might result in a higher synaptic density in the blindnessV1 and spare other sensory connections such as thalamocortical connections and corticocortical connections [26–28], which could lead to the increased CT in the visual cortex. Finally, the reduction of GMV in the V1 in early blindness is also consistent with our previous VBM result [37], which reflected axonal degeneration secondary to the impaired visual pathway in the early blindness [38–40].

Using this more effective method than VBM, we could further attribute the atrophy of GMV in these areas of the EBA group to the reduced SA despite the increased CT, since CV can be approximated by multiplying CT by SA, so we could infer that the reduction in SA is greater than the increase in CT. According to the study of the cortical development in early childhood, it was reported that CT is developed earlier than SA. At the age of two, CT can reach 97% of adult values, while SA only 69%. The authors concluded that cortical growth after age 1 is mainly induced by increases in SA [41]. The reduction of SA in V1 in the EBAs implied that the loss the light stimulation lagged the SA development after age 1, whereas the loss of light has little impact on the neuron development of CT. In the light of the radial unit hypothesis, SA is defined by the number of cortical columns, while CT is determined by the number of neurons within a column [41]. Although the number of cortical columns declined, the number of neurons in each column increased in V1 in the EBAs that might attribute to the other sensory connections of the crossmodal plasticity. A few literatures have reported that the V1 cortex played an important role in various sensory cognitions (such as auditory and tactile sensation) in blind people, although visual stimulation is lacking [2, 4, 5]. In short, the early-blind patients showed various changes in the CV, CT, and SA in the V1, which reflected the different influence factors like axonal degeneration, disuse atrophy, neurodevelopment, and plasticity that played a diverse effect on these indicators.

The left middle frontal lobe approximately overlaps the frontal eye field, which may be a heterogeneous component of multiple extrastriate visual areas in charge of eye movements like the intentional saccade trigger [42] and smooth pursuit, showing reduced CV in EB. It should also be a subsequent change caused by vision loss.

### 4.2. Cortical Thickness Alteration in the Visual Cortex (Right Lingual Gyrus, LING.R, and Right Lateral Occipital Gyrus, LOG.R) and Visual-Related Areas outside the Occipital Cortex (SPL.L and STG.L)

In contrast to the previous report of [15, 17] the increased CT in the LING.L in the early-blind subjects, significantly decreased CT was found in the LING.R in this study. As is known, this cortical area related to visual object recognition function forms part of the ventral visual pathway [43, 44]. Kim and Zatorre [45] found that this cortical area of a right hemisphere advantage might be recruited and activated to process auditory spatial position tasks in the blind during identifying auditory objects. In addition, according to the studies by Park et al. and Anurova et al. [12, 17], the negative correlations were found between CT of the occipital cortex and functional activation during auditory localization task in EB. This might account for the decreased thickness of the LING.R in EB who have better crossmodal plasticity abilities to guide action related to auditory localization and spatial discrimination. Meanwhile, the decrease of the CT in the LING.R is consistent with the theory mentioned above, in which the synaptic density is largest in the first postnatal year and gradually drops to the adult level by synaptic pruning during the activation and development.

Moreover, in line with this, many previous studies [15] have shown that increased CT was also located in the higher-level visual association areas (BA 19) such as extrastriate occipital cortex (LOG.R), which plays an important role in the attention, feature extracting, shape recognition, and multimodal integrating functions [46–52].

After visual deprivation, the alteration of the cortical thickness not only was found in the occipital cortex but also in the visual-related areas outside the occipital cortex, such as SPL.L and STG.L.

As a part of the dorsal attention network [53], the SPL is involved in spatial cognition [2, 54], sensorimotor, attention, and working memory [55–59]. There is a massive experience-related reorganization that blind people participate in the processing of spatial information from auditory [45, 60] and tactile [44] sense instead of visual input. According to a study by Park et al. and Anurova et al. [12, 17], areas with a thinner cortex should be associated with more effective function in EB. Therefore, the thin cortex of SPL may reveal that stronger plasticity of function in EB remaps the additional available sensory inputs (e.g., audition, touch) so that they could handle nonvisual tasks better in the absence of vision.

Our studies found that the increased CT in the left posterior STG (pSTG) is inconsistent with previous reports [12, 17] of the decreased CT in the anterior STG (aSTG). The STG is known to involve in the auditory comprehension process [61, 62]. Moreover, previous studies [63] have suggested that pSTG was more involved in the sound localization, while aSTG was related to auditory pattern processing, which may be explained by anterior-posterior functional dissociation in temporal areas. The increase of pSTG might correspond to the improvement of the ability of the sound localization in EBAs, which might indicate that cortical plasticity following visual deprivation enhances intramodal organization of auditory and tactile perception [64].

In summary, this study explored the structural characteristics of brain gray matter from different perspectives in early EBAs, including CV, SA, and CT. On the one hand, for V1, EBAs had significantly decreased CV and SA but increased CT; axonal degeneration is a key factor leading to a reduced SA of the V1, as well as neurodevelopmental retardation caused by visual loss, while the increase of CT results from the reduction of synaptic pruning due to neurodevelopmental retardation and crossmodal plasticity companying auditory and tactile perception; thus, the decrease of CV might indicate that the reduction of the SA was much more than the increase of the CT. On the other hand, visual-related areas outside the occipital cortex included the decrease of the CV in the left rostral middle frontal gyrus (r-MFC), the increase of the left superior temporal gyrus (STG.L), and the decrease of the CT in the left superior parietal lobule (SPL.L) and the right lingual gyrus (LING.R), which might mainly attribute to intramodal plasticity. Moreover, these cortical alterations were not correlated with the blindness duration. These structural findings might provide the certain basis to interpret the functions of brain regions in the blind. It has proved that the visual-related cortex does not shrink and degenerate and they are involved in various sensory cognitions to different degrees, which ignites a little light to achieve the possibility of crossmodal sensory substitution for blindness.

### 4.3. Limitation

The major limitation of this study is the limited small sample size, and further studies with larger cohorts are needed to explore different patterns between subgroups. Additionally, the results reveal structural changes, but the relationship between structural changes and functional activation has not been clearly understood and calls for further study for mutual interaction between structure and function.

## 5. Conclusion

Using the surface-based morphometric (SBM) method and combining different dimensions, such as CT, CV, and SA, to analyze the different morphological alterations of brain after visual deprivation, we investigated the cortical structural reorganization which occurs not only in the occipital cortex but also in the visual-related areas outside the occipital cortex. Our result demonstrated that structural reorganization of different brain regions in EBAs differently was influenced by the major factor(s) (neurodevelopment and/or degeneration and/or plasticity) and their interactions during development.

## Figures and Tables

**Table 1:** The significant altered cortical morphometry regions in EBA when compared with the NSC group.

Cortical morphometrics	Brain regions	Max	VtxMax	Size (mm^2^)	MNIX	MNIY	MNIZ	CWP	NVtxs
CT of left hemisphere	Superior parietal lobule	-5.693	19424	65.24	-26.9	-52.6	62.4	0.0002	131
	Cuneus	5.055	68981	64.45	-4.6	-70.3	13.7	0.0002	80
	Pericalcarine	3.454	112523	44.64	-8.6	-92.5	7.4	0.01157	44
	Lingual	3.635	56056	41.46	-16.7	-87.6	-8.3	0.01931	42
	Pericalcarine	4.816	28560	38.47	-23.8	-68.9	7.6	0.03332	97
	Superior temporal gyrus	4.332	84916	36.35	-60.4	-14.8	-3	0.04996	74
CT of right hemisphere	Lateral occipital gyrus	4.3	136992	50.41	13.2	-100.6	3.7	0.0042	68
	Lingual	-4.572	148749	39.79	25.8	-46.8	-6.4	0.02761	69
CV of left hemisphere	Pericalcarine	-5.638	75563	137.23	-14.8	-81.3	7.4	0.0002	264
	Pericalcarine	-3.068	29579	11.77	-13.3	-85.8	7.1	0.0014	21
	Rostral middle frontal gyrus	-3.424	4470	11.23	-32	31.7	34.9	0.0016	15
CV of right hemisphere	Pericalcarine	-4.575	86494	11.35	15.1	-75	6.4	0.0026	18
	Pericalcarine	-4.192	61382	8.49	7	-84.1	8.4	0.02997	11
SA of left hemisphere	Pericalcarine	-5.796	128114	155.1	-12.1	-77.7	3.7	0.0002	260
	Pericalcarine	-5.497	158064	146.1	-21.1	-71.3	7.8	0.0002	291
	Pericalcarine	-5.581	41148	70.84	-13.6	-90.8	3	0.0002	86
	Pericalcarine	-6.195	39176	52.31	-6.1	-91.9	7.8	0.0002	55
	Pericalcarine	-4.742	126455	41.71	-7.1	-85.4	11.1	0.0002	56
	Lingual	-5.064	162439	21.18	-26.3	-65.3	2.4	0.0002	56
	Cuneus	-5.263	158481	17.78	-4.9	-91.6	8.4	0.0002	19
	Lingual	-4.92	17525	16.56	-4.1	-83.3	-0.8	0.0002	23
	Cuneus	-3.621	135774	15.94	-3.7	-77.8	13	0.0002	23
	Cuneus	-3.461	39164	11.3	-4.7	-88.4	17.8	0.0016	13
	Cuneus	-4.573	68974	9.96	-7.1	-71.3	16	0.00519	14
	Lingual	-3.504	114632	9.52	-7.7	-84	-5.2	0.00878	7
	Lingual	-5.023	162465	8.44	-16.4	-64.3	0.4	0.02662	13
	Lingual	-3.355	139407	7.99	-21.6	-62.4	-0.1	0.04528	14
SA of right hemisphere	Pericalcarine	-4.964	25443	99.08	12.4	-88	1.3	0.0002	141
	Pericalcarine	-5.676	161917	90.23	6.7	-80.6	1.4	0.0002	112
	Lingual	-5.595	132374	68.78	16.4	-69.2	3.7	0.0002	104
	Pericalcarine	-6.278	86238	66.94	13.8	-85.9	8.1	0.0002	90
	Pericalcarine	-4.442	27615	23.38	8.8	-84	8.9	0.0002	30
	Pericalcarine	-5.256	135388	15.09	24.3	-66.1	7.6	0.0002	35
	Pericalcarine	-5.472	43531	13.78	19	-70.7	10.2	0.0004	26
	Lingual	-4.629	35860	12.7	5.3	-67.9	6.1	0.0006	23
	Lingual	-4.082	22548	9.47	18.6	-80.2	-12.1	0.01355	8
	Lingual	-3.719	90574	8.94	8.4	-77.4	-1.8	0.0199	8
	Pericalcarine	-4.312	156632	8.9	13.6	-91.7	7.1	0.0203	15
	Pericalcarine	-3.263	118284	8.65	16.1	-94.8	3.4	0.02642	10
	Lingual	-3.418	95382	8.63	8.3	-61.5	1.3	0.02662	16

Abbreviations: EBA: early-blind adolescents; NSC: normal-sighted controls; CT: cortical thickness; CV: cortical volume; SA: surface area; MNIX, Y, Z:the MNI coordinate of peak vertex; VtsMax: number of peak vertex of the significant cluster; CWP: cluster-wise probability and the nominal *P* value; NVtxs: number of vertices in cluster.

## Data Availability

The processed data required to reproduce these findings cannot be shared at this time as the data also forms part of an ongoing study.
